# Good and Bad Research Collaborations: Researchers’ Views on Science and Ethics in Global Health Research

**DOI:** 10.1371/journal.pone.0163579

**Published:** 2016-10-13

**Authors:** Michael Parker, Patricia Kingori

**Affiliations:** The Ethox Centre, Nuffield Department of Population Health, University of Oxford, Old Road Campus, Oxford, United Kingdom; Dafra Pharma, BELGIUM

## Abstract

There has been a dramatic rise in the scale and scope of collaborative global health research. A number of structural and scientific factors explain this growth and there has been much discussion of these in the literature. Little, if any, attention has been paid, however, to the factors identified by scientists and other research actors as important to successful research collaboration. This is surprising given that their decisions are likely to play a key role in the sustainability and effectiveness of global health research initiatives. In this paper, we report on qualitative research with leading scientists involved in major international research collaborations about their views on good and bad collaborations and the factors that inform their decision-making about joining and participating actively in research networks. We identify and discuss eight factors that researchers see as essential in judging the merits of active participation in global health research collaborations: opportunities for active involvement in cutting-edge, interesting science; effective leadership; competence of potential partners in and commitment to good scientific practice; capacity building; respect for the needs, interests and agendas of partners; opportunities for discussion and disagreement; trust and confidence; and, justice and fairness in collaboration. Our findings suggest that the sustainability and effectiveness of global health research collaborations has an important ethical or moral dimension for the research actors involved.

## Introduction

The idea that researchers should work in collaboration, rather than in isolation, has significantly reconfigured medical research. Reflecting a wider trend in scientific research since the Second World War, the scale and scope of global health research has grown dramatically in response to calls for more efficient and collaborative knowledge production [1, 2]. From the late 1990s and early 2000s, additional impetus has been given to these developments by claims that global health research collaborations have the potential to play a significant role in addressing global health disparities [3, 4]. The growth in global health research collaborations has also been driven by scientific developments, including those in genomics and in the development of statistical techniques and technological requirements for the analysis of very large datasets, and by major funding initiatives such as those of the Bill and Melinda Gates Foundation, and the Wellcome Trust [5].

The rapid growth in global health research networks involving complex historical, political and economic partnerships between researchers has been accompanied by literature addressing the ethical issues arising in global health research. Much of this literature has tended to focus on practical ethical concerns in operationalising international guidelines related to valid consent, standards of care, post-trial access, acceptable levels of risk, benefit sharing, and so on. There is also an emerging literature on the sociological and political aspects of such research collaborations primarily focused on understanding how macro level collaborations function [6, 7, 8, 9, 10]. The starting point for these accounts is a view that simply measuring the growth of collaborations provides little insight into their qualities, or whether they are equal or fair [11]. This literature has tended to suggest that research collaborations reproduce relationships and conditions which disproportionately favour high-income countries and institutions [12, 13]. Publication authorship, the named principal investigators and grant holders for funding applications, staff remuneration policies, tax exemption for foreign researchers and the ownership of samples and data have all been presented as areas where current inequalities undermine equal partnerships and collaborations [14, 15, 16]. It has been argued, that the term ‘collaboration’ can sometimes mask or exacerbate such problems [17, 18]. For instance, a recent examination of publications, resulting from global health research collaborations between American and European scientists and those in Central Africa, found that the most frequent role for African scientists was in providing samples and conducting fieldwork not research design [19]. These debates have been accompanied by a growing literature on the role of ‘ethics governance’ in ensuring high ethical standards in global health research collaborations [20, 21, 22].

Such insights have highlighted the importance of distinguishing between well-managed collaborations and ethically sound collaborations and have prompted calls for changes to the criteria for evaluating and assessing the fair and ethical conduct of such collaborations, such as the extent to which the collaboration provides tangible and timely resources to Global South collaborators and opportunities to be involved in areas of science which can provide opportunities for developing and strengthening new skillsets [20]. This academic debate has been mirrored in a wider policy and political debate about what might constitute the responsible conduct of collaborative research. At the level of guidance and policy, a number of initiatives have attempted to characterise ‘good’ collaborative research practice. These include: ‘Responsible Conduct in the Global Research Enterprise’ [21]; ‘Where there is no lawyer: Guidance for fairer contract negotiation in collaborative research partnerships’ [22]; ‘The Montreal Statement on Research Integrity in Cross-Boundary Research Collaborations’ [23]; ‘The COHRED Fairness Index for international collaborative partnerships [24].

What is striking about these academic debates and policy-level initiatives and guidelines, is that very little attention has been paid to capturing the experiences, values and attitudes of research actors directly involved in such collaborations or to how these factors influence perceptions of and judgements about whether to participate in them. Furthermore, they rarely involve consideration of researchers’ perspectives or evidence about the criteria they themselves employ to assess potential collaborations. As a consequence, there are few accounts of researchers acting as agents, in either constructing their own assessments and evaluations of collaborations or, actively choosing who they collaborate with on that basis. This is an important omission as an understanding of the judgements researchers make about whether to join or to contribute actively to global health research collaborations is going to be a major factor in understanding the requirements for successful, sustainable science. It is vital for research funders, researchers, and policymakers to have a good understanding of the experiences of research actors involved in global health and how they reach decisions about collaborations. As a first step towards addressing this gap, we report here on our analysis of a series of interviews conducted with research actors and funders involved in several high-profile international global health research networks.

## Methods

The findings reported in this paper are based on 22 qualitative interviews conducted with research actors over a two-year period. Those interviewed were purposively selected because they play a significant role in at least one—usually several—large international collaborative networks involving partners in both high and low income countries. The term ‘large international collaborative network’ here refers to collaborations involving partners in many i.e. more than 10—sometimes more than 20—countries across both low and high income settings. Interviewees were selected by their role and ranking in research. Using a snowball approach to sampling, a concerted effort was made to include a range of people playing roles of key importance in collaborative research. These included: principal investigators, research funders, network coordinators, clinical trial managers, epidemiologists, laboratory and sample managers, IT specialists, database managers, statisticians, clinical researchers and managers of research and health institutions. The number of principal investigators interviewed was 11. Interviewees self-identified as being from South East Asia, South Asia, East Africa, West Africa, Europe, Oceania, and North America. Each quotation in this paper is followed by a note of the role, self-identified geographical region of origin and current primary location on the interviewee. It is noteworthy, however, that current primary location was in practice often difficult to nominate because the roles played were often very mobile. A researcher might, for example, work and spend significant time in and even consider themselves to live in, more than one location. It is also necessary to note that congruence between region of origin and location of employment does not mean people are working in their country of origin: ‘West African Principal Investigator, West Africa’ might, for example, signify a Ghanaian researcher working as a PI in The Gambia. Attention was also paid to the gender of those interviewed; nine of the interviewees were female, thirteen were male. Gender is not associated with individual quotations in this paper in order to protect the confidentiality of the participants.

Following an in-depth discussion about the aims of the study, data management, and the use of anonymised interview extracts in publications, participants provided oral consent to participate. As is common in interviewing senior professionals, oral consent was deemed appropriate because the interviewees were senior and experienced. Consent was recorded before interviews started and is included in transcripts. This study, including the approach to consent, was approved by the Oxford University Tropical Medicine Research Ethics Committee (Ref: 020–06). Face-to-face interviews were conducted by MP in locations convenient to the interviewees. The interviews were about an hour in length. All but two were audio recorded and transcribed verbatim. One interview was conducted by email in French via a translator with key themes verified and expanded upon in person at a later date. The other was not recorded because of problems with the recording device, but detailed notes including verbatim quotations were taken during the interview. The interviews covered a range of subjects, some of these were initiated by the interviewees and some were informed by a topic guide which focused on the following areas: personal research career and experience of collaboration; experiences of deciding for/against collaboration; views about what makes the difference between effective and ineffective research collaborations; and factors of particular importance in collaborations between high and low-income countries.

Analysis was conducted in tandem with data collection, with initial analyses of early interviews informing the themes explored in those that followed. Following the completion of the interviews, analysis was subsequently conducted in a number of stages. The interview transcripts were read through by MP and PK and broad themes identified by each author. These themes were discussed and formed the basis of more in-depth line-by-line analysis of the transcripts. This was conducted and managed in NVIVO (Version 10). New themes identified in more detailed analysis were used to inform the analysis. An example of a broad theme was “Rule of collaborations” with “Obligations”, “Sticking to the rules” and “Breaking the rules” identified as sub-themes. This analysis was also informed by the literature on this subject and in particular the social science, ethics and medicine publications examining collaborations, partnerships and networks.

## Findings

[…] I get invited to participate in many things and in some cases I do kind of turn down the invitation or drag my feet somehow. It mainly depends on you know [if] the science is something [worth doing]. I need to be convinced that it’s worthwhile.

(West African Principal Investigator, West Africa)

As the number, scale and complexity of research collaborations have increased, individual researchers, such as the informant quoted above, have experienced more frequent invitations to join them. For busy researchers—short of time, energy and other resources—this means that decisions, which are sometimes difficult, need to be made quickly about which invitations to accept. Box 1 below sets out a summary list of key factors identified in our research as influencing researchers’ decisions about initiating, joining, remaining in, and actively contributing to research networks and their judgements of such collaborations as ‘good’ or ‘bad’. We expand on each of these at greater length below. However, it is worth noting here at the outset that while there are important differences and potential tensions between these criteria for our interviewees, they are also seen as interconnected in the making of their overall assessment of whether a collaboration was deemed to be ‘good’, where the term ‘good’ is seen to encompass both instrumental and intrinsic considerations.

**Box 1. Criteria for used by researchers in assessing collaborations**

- *Active involvement in cutting-edge*, *interesting science*
- Effective Leadership
- Competence in and commitment to good scientific practice
- Capacity building
- *Respect for the needs*, *interests and agendas of all partners*
- Opportunities for discussion and disagreement
- Trust and confidence
- Justice and fairness in collaboration

### 1. Active involvement in cutting-edge, interesting science

It was clear from all of those interviewed that the perceived quality and social value of the science being proposed was an important factor in their decisions about whether or not to join a collaboration. Whilst this was necessary for good collaboration it was rarely sufficient. A significant additional criterion for the researchers we spoke to in their assessment was whether the proposed collaboration offered them an opportunity to be *actively involved personally* in cutting-edge, interesting and outstanding science.

For researchers in low-income settings, worries of this kind were common and often grounded in personal experience, or in that of colleagues, of having been relegated to the role of “*a glorified field worker” (East African Principal Investigator*, *East Africa)*. That is, of being seen as responsible for providing samples but being excluded from the creative, interesting and ‘scientific’ features of the collaboration. As the following quote, from another African Principal Investigator illustrates, worries of this kind can be a major factor in deciding whether or not a collaboration is worth joining:

…one of the things that kind of makes me a feel a bit uncomfortable about joining …is when you feel that [collaborations] are really about getting access to samples without really having any kind of contribution […] And I think that’s something that I’d be wary about, where I felt **am I really going to contribute anything, you know, intellectually?**

(African Principal Investigator, East Africa)

As this quote illustrates, where it is believed that invitations to join collaborations are judged to be primarily for access to samples or motivated by tokenistic reasons—such as to give the appearance to funders that researchers from a particular region were involved—these were considered ‘bad’ collaborations by those researchers we interviewed. An additional implication of this worry was that, in instances where researchers felt that collaborations were too large, or centralised for them to have any realistic prospect of making a meaningful contribution or in cases where they believed for other reasons they were being invited to participate for reasons unrelated to their scientific contribution, researchers were likely to refuse or to participate reluctantly. All the researchers interviewed valued being included in creative science and the potential for involvement of this type was foremost in their minds when evaluating the merits of collaboration.

### 2. Effective Leadership

Notwithstanding the importance of active personal involvement in the setting of scientific agendas, great emphasis was also placed on the value of effective overarching leadership of the collaborative project or network. Generally, in our interviews, this took the form of an acknowledgement of the key role played by an individual with overall vision for the collaboration or international network. The involvement of an effective leader was also a common reason given for accepting an invitation to be involved in collaboration. A good leader was someone highly respected in their field and someone with an impressive scientific vision:

I have to say she was always way ahead of us in her thinking in the area but we have always admired her…

(American Principal Investigator, East Africa)

It was also clear, that in addition to his or her intellectual leadership the leader had to be someone who was genuinely interested in the relationships underpinning the collaboration. This was seen as very important in developing trust among collaborators. The following quote illustrates the complementarity of these two characteristics:

Yeah, clear leadership, he provides it, everyone else follows which is…The best form of governance is benevolent dictatorship and I think that's probably slightly controversial but I think that's what [is required] they trust him, he evidently cares…he cares about that relationship and the network… I think that's probably one of the reasons why it has worked so well.

(European Network Coordinator, Europe)

What this quote suggests is that where the collaboration is led by an effective leader, who is strongly committed to the maintaining of trusting, respectful relationships, this need not be in tension with—and may in fact be essential for ensuring—the active involvement of partners in resource-poor settings in interesting, cutting-edge science.

In addition to the importance of overarching network leadership, effective leadership was also seen as an important characteristic at individual partner institutions, for example where these were under consideration as potential partners. This clinical trials manager suggests that:

…[a good] site, a good collaborator, or a good collaborator with his team, or her team, will be somebody with good leadership skills and can set up a team that can deliver.

(Asian Clinical Trials Manager, South East Asia)

This informant argues that a good collaborator is someone who can ‘deliver’ and this is seen to call for someone with good leadership skills. Such judgements were not always or only about particular individuals. Notions of a good institution, research team and leader were often used interchangeably and underpinning such assessments was the ability to deliver good data in a timely manner.

In summary, when making assessments about a potential or current collaboration, whether from the perspective of a researcher in a low-income country being invited to participate or from that of a senior researcher considering potential partners, the type of leader involved was deemed important. Those interviewed emphasised the importance of leaders who were engaged in and making, a valuable contribution to their subject area. In addition, a good leader was interested in and cared about the details of the collaboration and ultimately is someone capable of ensuring the timely delivery of good data. It is important to note that the judgement being made here is one that has both a scientific and an ethical or moral component.

### 3. Competence in and commitment to good scientific practice

Given the importance placed on scientific value and good leadership, and their close connection to the concept of a person or institution that is able to ‘deliver’, it is perhaps unsurprising that researchers also emphasised the scientific competence of potential collaborators and their perceived commitment to good scientific practice as key factors in judging whether a collaboration or collaborator would be likely to be a good or bad one. It is also unsurprising that in their assessments of competence and commitment to scientific practice researchers tended to emphasise ‘deliverability’ and ‘timeliness’ as signs of a good collaborator and collaboration.

Our interviewees argued that good collaborators were those who were, or were likely to be, able to deliver data, samples and analysis in a timely way to enable research to proceed smoothly. In the quote below, for example, a clinical trials manager describes having entered into a collaboration agreement with a research team only to find that the collaborator had not been able to meet their expected recruitment timelines:

Interviewee: They […] got their approvals later than anyone else and once they got approval they recruited one patient and that was about 9 months ago.

Interviewer: And how many were you hoping for?

Interviewee: 120.

(Asian Clinical Trials Manager, South East Asia)

After giving this example, the trials manager went on to elaborate on the factors informing their view of a good collaborator, contrasting the group mentioned in the quote above with another who was judged to be a ‘good’ collaborator despite working under difficult conditions:

…a good collaborator, a good team, an example would be you know the [..] site that I told you about…now that’s a good collaborative site because the team is really good, they have the patients, malaria is the problem for them. So although it’s very difficult to get to and they are in [a distant location], **we like them as a site because they’re very productive, they deliver.**

(Asian Clinical Trials Manager, South East Asia)

It is worth noting that the term ‘good’ is being used in the quote above, and in other interviews, in both instrumental and moral senses. Instrumentally, the ‘good’ collaborator is productive and delivers data. Here the concern is about good scientific practice during a collaboration. Morally, a collaborator’s ability to collect data: *“[…]in a structured way and enter[ed] into a computer system and analyse[d]…”* (European Principal Investigator, Europe) indicated that they respected their role and the time of colleagues. Similarly, it was a bad collaborator in both senses—scientific and moral—who sent samples in *“inappropriate packaging*, *not cool*, *melted*, *[with] lids…off”* (European Laboratory Manager, Europe) because this meant a huge amount of salvage work and achieving research aims more difficult. Hence, having samples—and data—delivered on time and in a condition which followed procedures indicated that the collaboration’s aims and objectives were being respected and that ultimately an individual or team was good, in the sense that they took their responsibilities seriously and respected colleagues.

The use of ‘ability to deliver’ and ‘compliance with standard procedures’ as criteria for a good collaborator was not seen as unproblematic, however, particularly, but not exclusively, by researchers in low-income settings. This was because this assessment often omitted consideration of real world challenges in conducting research. These included significant external constraints which meant that even highly committed and responsible researchers were likely to experience difficulties in following procedures. As an illustrative example, a PI working in a low-income setting describes how practical and logistical concerns shape the timely delivery of data and its condition in at the point of arrival at a biorepository. She explains that:

…when you operate from Africa you don’t control the time where you sample has to go because […] the few flights that are available, not all of them would accept certain a type of material [e.g] DNA. We’ve been trying to ship this thing for nearly 45 days and in the morning the guy calls and says, “Hey I found a spot on [the next flight]. If you can get your samples ready in an hour it will go today, otherwise we’re going to have to wait one week.” […] so the guy came … he saw us we were frantically getting the things together…but the goods were gone that day.

(African Principal Investigator, West Africa)

In situations where samples have not have been delivered according to procedure or are late, there can be external mitigating factors which have meant that compromises have had to be made between timeliness and orderliness. It noteworthy that these factors were widely acknowledged by the research actors we interviewed, wherever they came from. For this reason, many researchers mentioned that they did not make assessments of their collaborators in low-income contexts based solely on deliverability and timeliness. These judgements were multifaceted. It was clear however that having ‘the right attitude’ was very important for all involved. Worries about this were expressed in a variety of ways. For example, one researcher, a laboratory manager in a high-income country, who was very aware of and understanding about the challenges facing researchers in low-income settings, was nonetheless very unhappy about,

[being treated like] they're doing you a favour by having sent you what they've sent you…

(European Laboratory Manager, Europe)

despite the significant increased workload this caused for those receiving and curating the samples.

### 4. Capacity building

In addition to the opportunities it might provide for personal involvement in cutting edge science, senior researchers in low-income settings also placed a great deal of emphasis on the importance of ‘capacity building’ for others as a key component of a good collaboration. For all of those we interviewed, capacity building was considered crucial, and its absence in collaborations viewed very negatively indeed. The term ‘capacity building’ was employed in different ways to refer to activities including training courses or fellowship roles for early-career researchers, studentship funding opportunities for PhD students, and the provision of scientific equipment that might have uses beyond the project itself. Broadly speaking, capacity building was understood as the potential for opportunities to increase scientific competence and expertise of both experienced and younger scientists, to gain locally important added value more generally from participating in research. In this quote, a PI describes the anticipated benefits of an imminent collaboration:

It will expose us all to opportunities of training … that we wouldn’t necessarily be able to get a hold of by ourselves because we’re not part of that community [the collaboration]. So I think it’s got huge spin off [in] educational and intellectual academic benefits for me and for lots of other people.

(European Principal Investigator, East Africa)

For this researcher, capacity building was understood to include both training opportunities for individual researchers and wider anticipated educational benefits for their research centre. Indeed, among those interviewed, capacity building was often viewed as a cluster of benefits. In the quote below, for example, a PI suggests that capacity building might include both the achievement of internationally recognised standards and access to new technologies:

So what made us decide to take part in the […] project was the desire to link up with other teams, to standardise our practice with international norms, to benefit from new technologies, and to increase our capacity so that we could produce better results from all the work we had done [previously].

*(African Principal Investigator*, West Africa)

Taken together these quotes illustrate how capacity building, access to educational opportunities and new technologies were seen as essential short and long-term goals for researchers. Whilst it was clear that in the short-term these resources might be required for researchers to meet current project specific aims and procedures, the longer term impact was also seen as pertinent in discerning whether a collaboration could be considered good. There was an expectation that capacity building would provide wider and more far-reaching long-term benefits and greater equality for research teams, institutions and, in some cases, at a national level.

Notwithstanding the importance of recognising differences in capacity, it was also clear that interviewees believed that requiring high standards, particularly of early-career researchers, was crucial to the long-term development of researchers:

…what happens I think with fellows from, you know for instance within the network is that they [say] “Oh they’re African fellows so you know their starting point is really, really low so we need to give them a bit more time, we shouldn’t expect too much, shouldn’t give them pressure. And what that does is that **it just perpetuates the whole thing because you know if you expect, if your expectations are low then […] you’ll have less,** but if you push people and say… whether they like it or not they have to get up to scratch…

(African Principal Investigator, East Africa)

This East African PI was objecting not only to what was judged to be a condescending attitude among some researchers involved in capacity building activities, who accepted lower standards from researchers operating in some contexts but also because it was seen as an attitude that reduced the likelihood of producing high-quality independent researchers in the long-term. This suggests that further to the availability of training and other more tangible benefits such as equipment, the attitudes of collaborators to early-career research also deemed crucial to the success or otherwise of capacity-building.

Here again, the emphasis placed on capacity-building by those responsible for the overall leadership of the collaboration and their attitude towards young scientists was viewed as a crucial indicator of good leadership. Here, a research coordinator relates the success of a particular collaborative network to the leader’s explicit and widely known commitment to both capacity-building and high standards:

…she [the leader of the collaboration] cares about… bringing on this group of younger African and Asian researchers, she evidently does care about that…

(European Network Coordinator, Europe)

### 5. Respect for the needs, interests and agendas of all collaborators

All those interviewed recognised that whilst participation in large collaborations could potentially produce important benefits both for themselves and for global health, their realisation in practice requires all collaborators to exhibit flexibility and willingness to compromise:

…we’re all investigators, we’re all used to doing things in certain ways and sometimes …the group might decide to do things a bit different. […] **even if you don’t like [it] you sort of accept, you change your way of doing things so that you can fit in the group**…

(African Principal Investigator, West Africa)

At the same time, there were concerns that compromises and flexibility should be fair and should not further exacerbate unequal relationships between the collaborators. The importance of maintaining and promoting equity whilst making compromises was a key theme for all but it was inevitably of particular concern to those working in low-income settings. For many interviewed, the issue was not whether compromises were necessary but the extent and distribution of the compromises and ultimately which collaborators were required to compromise most, and whether this was reasonable. In the following quote a European PI working in East Africa suggests that most collaborations have yet to resolve this tension in ways that address the agendas and interests of African scientists:

…actually not that much has changed […] **the relationship between the North and the South is still exactly as it was, the North has money and tries to dictate the research agenda.** Africans are continually trying to respond to somebody else's agenda whilst having their own agenda at the same time which I think very often doesn't get investigated…

(European Principal Investigator, East Africa)

Notwithstanding the very great importance placed on mutual respect and equality in collaborations by most interviewees, it was clear that any attempt to pretend that things are more equal than they are should be resisted. This PI explains why pretending there is equality is detrimental to collaborations:

…respect is recognising that you have this strength […] that you are bringing to the table, and I will respect you because of that. I’m not going to pretend that you’re equal … **it’s almost like by pretending that we’re equal is more of an insult.** By pretending… it’s equal partnership and we care what you, what you think and whatever I think that that’s, you know **that is quite insulting because I know that I’m not really contributing to it**… it’s those subtle things that I feel that are more, are a lot more difficult to address … **you know [it’s] uncomfortable so it’s better just to pretend that it’s all you know “Oh I really respect you, I really value what it is that you’re saying blah, blah, blah…”**

(African Principal Investigator, East Africa)

This quote also highlights the importance placed on non-consequentialist moral considerations, in this case, mutual respect. This is non-consequentialist because it is seen as morally required even if it would be easier for everyone to avoid the issue. This related to a wider view that, for some researchers, respect meant having an open and honest discussion and not pretending or being condescending about the properties and qualities of their collaboration.

### 6. Opportunities for discussion and disagreement

The complexity, diversity and distribution of collaborative global health networks means that effective communication is a crucial factor in distinguishing between good and bad collaborations. All interviewees were able to give examples of both effective and ineffective communication. Some examples concerned the role of good communication in the effective day-to-day practical conduct of science. In this quote a PI provides an example:

I think it is much better we meet each other and these things are like brought out into the open and thrashed out …

(Asian Principal Investigator, South Asia)

Other examples related more closely to the concerns above, for instance to the importance of communicating various needs and motivations as a way of ensuring these are addressed and that there is ‘buy-in’ to the project. Here, a PI explains the importance of being open and taking seriously the motivations for being involved in the collaboration:

I suppose what you have to do is you have to, once you've got your network together and everybody is signed up you have to then **have a very open discussion about what do you want to get out of it. What’s your motivation? What do you want to get out of it at the end? And you have to really listen to what people say and take seriously** …then you have to say okay so we have to really help you to do that.

(European Principal Investigator, East Africa)

Several interviewees expressed a belief that openness was key to sustainable collaboration because it was a crucial for accepting and living with decisions. In the following quote a PI characterises their current collaboration as being ‘good’ because of its emphasis on communication between the collaborators:

…the really important things that I’ve noticed in this network since the beginning is the openness, everything is put on the table, everything is discussed and then we basically decide what to do. …**being able to discuss things openly it really decreases the chances of some people being frustrated because they haven’t had chance to voice their views.** Even if their views are not taken into account they get a chance to voice it and you know they got bitten, then they will swallow it, they’ll move on.

(African Principal Investigator, West Africa)

Finally, echoing and further illustrating a point made above, a lot of emphasis was placed on the importance of open and frank discussion of disparities of resources and capacity as key to effective collaboration even when the subject matter is difficult or uncomfortable:

things … have to be kind of acknowledged by both sides and trust and talked about in a very, very open way and I don’t think is something that is ever discussed really, really between partners […] it’s one of those things that you think **oh it’s better not to go there because it’s just going to come across as being patronising or racist or whatever so let’s not address the fact that there’s a disparity in the capacity** or there’s a disparity in terms of contribution let’s just concentrate on what it is that we’re going to do.

(African Principal Investigator, East Africa)

Open discussion was a prominent criterion in assessments of a good collaboration because it meant that researchers had an opportunity to discuss their motivations for joining and what they wanted to achieve in addition to the goals of the collaboration. Furthermore, it signalled that all members were given the opportunity to have a voice, even when discussing difficult and uncomfortable issues. However, being able to do this required a certain degree of trust and confidence in the collaboration.

### 7. Trust and confidence

In addition to questions related to scientific value, and concerns about the potential benefits of collaboration, two other influential factors about research collaboration and views about the value of existing or previous collaborations were trust and confidence. From the perspective of the researchers interviewed, a great deal depends on views about the character and behaviour of potential collaborators and whether they could be trusted. For many researchers, trust in a collaborator was fostered by their *“reputation”* and the absence of the types of *“undesirable qualities”* (Asian Principal Investigator, South Asia) mentioned above. Here, another PI discusses the importance of such trust when deciding whether a potential collaborator is someone to whom samples might be sent:

…you have to send samples somewhere that you don’t know what's going to happen …what they're going to do with samples, I think that's really hard…**if I have to send my samples to somebody I have to know that people very well.**

(South East Asian Principal Investigator, Europe)

This idea of only feeling comfortable with sending samples to someone who is known to the researchers which was discussed by this PI makes it clear that building up trust required for effective collaboration can take time. In the quote below a representative from a funding body provides their perspective on the time taken to build trust between collaborators:

…there's a lot of t**rust that has built up over a long period of time and it's not simply about the money that the [Funder] can throw at these things and no amount of money can build in the innate kind of synergies** that need to coalesce to make for a fruitful collaboration…time is of the essence … and you can't sort of fast forward …I think there's a danger in that and I think it's all got to be timely in terms of the associations and how they develop.

(European Research Funder, Europe)

The primary focus of our research was on the relationships between potential scientific research partners and this inevitably meant that our interviews tended to focus on the practices and character traits of researchers and research institutions. However, many researchers also stressed the complementary importance of maintaining the trust of local communities in their work when considering possible research collaborations. This PI explains the significance of the trust of communities as follows:

Scientists in developing countries must be aware that their communities put their trust in them, and so they must be constantly alert to defend their interests, both moral and material.

(African Principal Investigator, West Africa)

This researcher explains that communities trust and expect that researchers will act in their best interests. This places a different emphasis on ideas of trust in research collaborations.

It was clear in the interviews, that interpersonal trust, whilst important, was not the only relevant question. In judgements about good or bad collaborations, considerations was also given to whether they could have confidence in the systems, institutions and practices involved to provide protections beyond those offered by trusted individuals. This was particularly valuable in situations where trust had not yet been established, for example, where potential research collaborators were not known personally prior to the approach. Descriptions of the systems not only included procedures related to data collection but also the ethical considerations and good practices of the collaboration. The following quote explains the significance of having these systems in place for effective collaboration and also for its sustainability in terms of funding and reputation:

[Funders are] nervous about funding a project that has anything dubious with ethics or science or anything else. So if they know there’s a network that has a whole series of standard practices, good practices then I think they will preferentially want to fund them over and beyond the quality of their science.

(European Research Network Manager, Europe)

Here, as before, it can be seen that ethical criteria such as ‘trust’ and ‘confidence’ had instrumental and financial implications for the conduct and viability of collaborations and ultimately for the production of scientific knowledge.

### 8. Justice and fairness in collaboration

Considerations of justice and fairness play a central role in the assessment of collaborations. Worries about fairness can arise in several different ways. A notable manifestation of unfairness for many interviewees concerned the lack of recognition of expertise and scientific roles of less visible partners. A key emphasis was on recognising interdependence of all partners:

There must be a fair exchange, and each side must recognise the importance of the other, in order to build trust between them. However outstanding the performance levels of laboratories in the north may be, without the quality of the data collected in the south, nothing could be achieved.

(African Principal Investigator, West Africa)

As this PI explains, ideas of justice and fairness were seen as vital factors in gaining and maintaining trust in collaborations. Key to justice and fairness, and hence to trust, was recognition of the mutual and cooperative nature of successful research. Northern researchers are not self-sufficient benefactors providing capacity-building and strengthening resources. They need the data, samples, skills, experience and expertise contributed by their Southern partners. Lack of recognition can have crucial implications for key scientific measures such as authorship and can become self-perpetuating. Taking authorship as an illustrative example, the following interviewee argues that many of the issues of fairness and justice in publications authorship relates to *“a failure of imagination”* to consider the range of actors and work involved in scientific knowledge production:

[…] I've come across the situation where a group of researchers in a laboratory may write the paper and they may [list] the authors and then I say "What about the people who provide the samples?" and they "Well but they didn't do any of the work". **They haven't conceived that there was work that happened before the samples entered into the laboratory and it's a failure of imagination […]** [It] maybe took a series of doctors working over a period of ten years’ time, working outside the health system in Africa, poorly funded, being up in the middle of the night, coming in when there's no generators, having to take five, you know five hours to get to the hospital because it's a long way away and they're, for many, many months they're collecting samples from patients and those get condensed down to samples.

(European Principal Investigator, Europe)

Importantly, concerns about appropriate recognition were not also expressed about the lack of recognition accorded to laboratory staff and analysts, whether in Northern or in Southern institutions:

I've been in the clinic and there's been enthusiastic clinicians who want to investigate a clinical problem and they send it off to the laboratory and they can see a bunch of people wearing white coats […] and they assume that those guys are, they're just the sort of technicians there and they, you know they, they don't sort of conceive that these people in the laboratory might have had to develop some new methodologies and apply a lot of creativity, maybe stay up in the middle of the night. **I mean often people don't give other people credit for the sleepless night, you know for a long time…I think if you're doing collaborative research it's, there's something of an obligation to see what happens on the other side of the divide** […].

(European Principal Investigator, Europe)

Taken together these quotes demonstrate the importance of inclusive, but fair and justified, authorship as a marker of justice and fairness in collaborations.

In addition to questions about justice and fairness in relation to markers of scientific contribution and its value, concerns about justice and fairness also focussed on the approach collaborations took to benefit sharing and the use of resources to benefit the local population (research participants, their kin and wider society). From this position, a good collaboration was sufficiently flexible to allow research partners to meet their obligations to the communities in which, and with whom, they conducted their research as this PI explains further:

… blood donor centres consistently provide meals for willing donors.** The issue here is not the amount of blood donated, it is a matter of principle and equity. […] In our own research, we have fought until we succeeded in getting schools built in both villages.** Each time we get a new grant we use some of it to support the community (providing food for the children’s school canteen; mills to grind meal for the women’s groups; financial aid for students at college).

(African Principal Investigator, West Africa)

Several examples were given in our interviews of situations where the absence of local benefits had been seen as a significant obstacle to collaboration. It is clear that issues relating to benefits are important to communities and also play a key role in the deliberations of research ethics committees and research institutions about the value of participating in research collaborations. It is also clear, however, that this is also a matter of personal morality for researchers both in low-income settings and for those in high-income settings who collaborate with them. In the following quote a PI explains this point further:

**I always feel uneasy when I have to do a study of a poor family (who may not be getting enough to eat, but must nevertheless give a blood sample); I have plenty of funds at my disposal** (for example we use an enormous 4x4 vehicle which costs more than the annual budget of the whole community) and yet I am told that we must not offer material aid (to participants) because that would be bribing them to take part in the study.

(African Principal Investigator, West Africa)

It is important to researchers, not only that they themselves should be respected for the contribution they make, but also that the societies in which they work should not be treated as a means to an end. Collaborations which were just and fair would be ones that paid appropriate attention and respect to the full range of actors involved in producing scientific knowledge.

## Discussion

Developments in scientific methods, funding, and policy have led to a rapid growth in the number and scale of international collaborative networks which bring together researchers in high and low income countries to address scientific questions in global health. This growth has led to the parallel emergence of a number of complex ethical problems not previously encountered in combination. A substantial bioethics and social science literature has grown around issues including: informed consent; social value and benefit sharing; community engagement; data-sharing; the collection and export of biological samples. Despite the growth of interest in the ethical implications of international research networks and their practical implications in low-income settings, however, surprisingly little attention has been paid to the relations between research collaborators in different locations and between globally distributed research institutions and funders. There have been some attempts at the level of policy to map out the requirements for ‘fair research collaborations’ [25, 26, 27] but little systematic academic research has been undertaken to explore the experiences of research actors themselves about collaboration, about what makes the difference between a good or bad collaboration or collaborator, or about the factors that influence their decisions about whether or not to join collaborative research networks when invited to do so—and about who to invite to join research collaborations they initiate.

As a first step towards addressing this gap in the literature, we interviewed a number of research actors with significant experience of participation in high profile collaborative global health research networks involving partners in low and high income countries. Half of our interviewees were principal investigators whose work involves the making of frequent decisions about potential collaborations and collaborators. Many of these interviewees are internationally recognised and well-known leading senior researchers in global health. The majority of the remaining interviewees were mid-career researchers likely to be making such judgements in the future, and those involved in the ‘management’ or funding of research, whose views and experiences are influential in such judgements.

Our analysis of these interviews has enabled us to identify eight key factors used by experienced research actors in making sense of the difference between good and bad research collaborations and in making decisions about whether or not to accept invitations to participate or about whom to invite to participate in collaborations they are initiating. These factors are listed in Box 1. The picture of research networks and of the collaborative production of scientific knowledge in global health that emerges from our analysis is a complex set of multiple relations and practices characterised by activities and concerns beyond the ‘scientific’ as commonly understood. Whilst this might to some extent be unsurprising for those with a social scientific background, a key finding here is that this is the picture of science that emerges *in the accounts of scientists themselves*. One of our main findings, therefore, is that the day-to-day conduct of collaborative research is, for experienced scientific researchers themselves, a complex interweaving of scientific, social, political and ethical concerns.

This interweaving of scientific, social, political and ethical concerns in our interviewees’ accounts of the collaborations they had experienced was both striking and unexpected. At several points in our analysis, we expected to be able to tease apart concerns relating to scientific practice from those to do with collaborative relationships and ethical practice but this proved difficult. The concerns of the researchers we spoke to and their views about collaborations suggest a complex interdependence between the scientific and the ethical even where those precise terms are not used. This finding is reflected in the title chosen for this paper, ‘‘Good and Bad Research Collaborations: researchers’ views on science and ethics in global health research”, and in our decision not to use the phrase ‘Ethical Research Collaborations’ in order to indicate that conceptions of ‘good and bad’ collaborations had both a scientific and an ethical component.

It is also apparent that the addressing of these concerns is seen by scientists as part of the day-to-day work required for collaborative global health research to be both successful and sustainable. What this suggests, is that the successful functioning of global health research networks and hence the successful production of scientific knowledge, is seen by scientists to require of them and of other research actors such as fieldworkers and laboratory staff, a great deal of what might perhaps be described as ‘moral work’ in addition to what might perhaps more commonly be thought of as ‘science’. Although none of our interviewees used or would use the term ‘moral work’ to describe what they do, it was clear in much of what we heard, that no neat distinction can be made between the scientific and the ethical in understanding what is required for good research collaboration. The conditions required for successful scientific collaboration and the production of useful, relevant scientific knowledge involve a complex interweaving of scientific, practical and moral practices, such as for example the building and maintaining of trust, paying careful attention to fairness in the recognition of efforts, ensuring that scientists in low-income settings are able to meet their obligations to local communities, and the promotion of mutual respect. This supports the findings of our research in other areas in which we have described the central role of ‘moral work’ and ‘moral craft’ in successful science and highlighted the fact that this work is undertaken by a wide range of research actors including not only leading internationally recognised scientists but also frontline research workers such as fieldworkers, laboratory staff, sample managers, health professionals, the managers and administrators of global health research networks, and the Directors of research institutions [28, 29, 30].

Although it was clear that for the majority of our interviewees all of the factors listed in Box 1 are important, there was also recognition that there were some situations in which ‘trade-offs’ had to be made between them. One example of this was in situations where collaborations which were less than ideal in many other respects might nevertheless be seen as addressing significant, locally-relevant health needs. All of the researchers we interviewed placed a very high priority on the value of high quality research into diseases affecting people living in low-income settings. They also recognised that they were required to conduct research in an imperfect world and it was clear from our interviews that researchers were willing to put up with a lot of inconvenience and imperfection to be involved in what they perceived to be good, useful science of value to the communities in which they conduct their research. Situations in which resources for capacity building were limited for example, might be tolerated to some degree, even though reluctantly, if the collaboration was seen to be addressing a worthy scientific question of local relevance.

While those interviewed were broadly in agreement about most of the factors contributing to successful collaborations, there were also key differences. Often these were difference of emphasis, for example a greater stress was placed on the importance of capacity building by some researchers than others. In some cases, however, the tensions were more profound. A good example of this was the extent to which researchers viewed context as a factor to be taken into account in the judgement of whether someone was or was not a good collaborator. It was recognised by all that geographical location and limited access to resources, presented significant challenges that could sometimes affect the ability of partners to meet deadlines. However, in discussions about capacity-building and training some researchers argued that all researchers should be measured by the same standard irrespective of their context and that not to do so could be perceived as being condescending and unhelpful. Others argued that levels of training and ability were highly contextualised and that allowances should be made. Such disagreements illustrate the complexities in constructing ‘good’ collaborations and also the difficultly addressing all the expectations of those involved.

Although our research did not set out to identify stable differences between interviewees with different characteristics such as gender, role, geographic location and so on it was interesting to note three themes that did appear to be expressed more forcefully by researchers in some roles. The first is that, as might be expected, concerns about whether or not a potential collaboration was going to provide opportunities to be actively involved in cutting-edge, interesting science were most strongly expressed by researchers from low-income settings who were often not the leaders of the collaborations in which they were involved. Relatedly, perhaps, it was also noteworthy that those who expressed enthusiasm for opportunities for open discussion and disagreement also tended to be based in low-income settings. Finally, those who were involved in the management and co-ordination of collaborations, usually but not always in high-income countries, were those who placed a great deal of emphasis on whether potential partners were competent in and committed to good, efficient scientific practice. None of the issues emerging in our analysis were raised solely by participants from a particular gender, geographical location, or role, however.

Given its relatively small-scale, this study inevitably has a number of limitations. Attention was paid to ensuring a range of different research roles and experiences and to geographical distribution and diversity of nationality and ethnicity. However, it was nonetheless a relatively small study. Those we interviewed were identified primarily through a snowball approach and this meant that they tended to be involved in, or have experience of, research collaborations of a similar, very large, global scale. Not all research collaborations are this large—many, probably the majority, are bilateral or have a relatively small number of partners. Another possible limitation is the fact although that the topic guides and interview questions were relatively neutral about what, for example, an appropriate answer might be to the question, ‘What for you are the most important differences between good and bad research collaborations?’, it is likely that interviewees would have seen the study as ‘social science’. This might possibly have excluded some very technical or traditionally ‘scientific’ answers to the question being provided. We are aware for example of the fact that successful collaborations in contemporary science require access to efficient broadband connections, sequencing facilities, biorepositories and skilled data analysts. Notwithstanding these limitations and the need for further research, this study does provide valuable evidence that, from the scientists’ own perspectives the sustainability and effectiveness of global health research collaborations has a crucial ethical or moral dimension and that this is seen as an important part of the day-to-day work of successful science.

Finally, it is important to note that the primary focus of this research and of this paper has been on the qualities and experiences of global health collaborations involving high and low-income country partners. Those interviewed were all reflecting on their roles in such collaborations. However, it is likely that many of the experiences and concerns presented here will be familiar to those engaged in other kinds of collaborations such as those involving partners in different high-income countries. As one of the reviewers of this paper helpfully pointed out, issues relating to leadership, acknowledgement, trust and the tensions between the ‘centre’ and ‘periphery’ of research networks such as worries about being seen as ‘merely sample collectors’ are a feature of many collaborations, not only those involving partners in low-income settings. Our aim in this paper has not primarily been to suggest that global health collaborations are uniquely different to other kinds of research collaborations but to investigate the experiences and values of those who involved in such collaborations. We acknowledge the importance of avoiding global health network ‘exceptionalism’ and see the investigation of similarities and differences between different forms of scientific collaboration as an important avenue for future research.

## Conclusion

Global health research is increasingly taking the form of large scale collaborations. There are a number of important scientific, technological, policy and economic reasons for this. Our motivation for conducting the pilot study described in this paper was our awareness of the lack of research exploring the experiences of those who are tasked with the building and maintenance of effective scientific collaborations on a day-to-day basis—the scientists, research managers, and laboratory staff themselves. Little if any attention has been paid to the factors that these and other research actors see as important to successful research collaboration and that inform their decision-making about which collaborations to join and which to refuse. Many of the factors influencing such judgements will inevitably be related to access to technologies, expertise, and sources of funding. However, the picture of contemporary global health science that emerges from our analysis is a complex set of multiple relations and practices characterised by activities and concerns beyond the ‘scientific’ as commonly understood. For scientists themselves, the day-to-day conduct of collaborative research is a complex interweaving of scientific, social, political and ethical concerns.
